# Predicting willingness to consume healthy brand foods using the theory of planned behavior: the role of nutritional literacy

**DOI:** 10.3389/fnut.2024.1353569

**Published:** 2024-03-22

**Authors:** Rony Francisco Chilón-Troncos, Elizabeth Emperatriz García-Salirrosas, Manuel Escobar-Farfán, Dany Yudet Millones-Liza, Miluska Villar-Guevara

**Affiliations:** ^1^Unidad de Ciencias Empresariales, Escuela de Posgrado, Universidad Peruana Unión, Lima, Peru; ^2^Faculty Management Science, Universidad Autónoma del Perú, Lima, Peru; ^3^Departamento de Administración, Facultad de Administración y Economía, Universidad de Santiago de Chile, Santiago, Chile; ^4^Escuela Profesional de Administración, Facultad de Ciencias Empresariales, Universidad Peruana Unión, Lima, Peru; ^5^Escuela Profesional de Administración, Facultad de Ciencias Empresariales, Universidad Peruana Unión, Juliaca, Peru

**Keywords:** nutrition literacy, attitude, subjective norm, perceived behavioral control, planned behavior, willingness to consume, healthy brand foods

## Abstract

**Introduction:**

The willingness to consume healthy foods has highlighted the growing importance of health, even more so when it comes to food choice, and predicting the willingness to consume foods of a healthy brand represents an action that leads to the practice of conscious eating habits, but what is behind this willingness? To answer this question and based on previous studies such as the theory of planned behavior and nutritional literacy, this study aimed to build a predictive model through an empirical study to examine the influence of nutritional literacy (NL) on attitude (ATT), subjective norm (SN) and perceived behavioral control (PBC), as well as to determine the influence of the three variables of the theory of planned behavior (TPB) on the willingness to consume healthy brand foods (WCHBF) in the Peruvian market.

**Methods:**

The research focused on the population that stated that they were consumers of the Unión brand (a brand whose value proposition is the sale of healthy foods), obtaining 482 consumers. The study was conducted under a quantitative, non-experimental, cross-sectional design approach.

**Results:**

The results support the existence of a positive and significant effect of NL on ATT, SN, and PBC, finding the exact behavior of SN and PBC in WCHBF; however, in the proposed model, it is observed that ATT has no impact on WCHBF.

**Conclusion:**

Applying strategies that lead to a change in consumer behavior towards healthy brands is a matter of time and will. In this context, the findings indicate that nutritional literacy plays an essential role in the willingness to consume healthy foods, which sheds more light on the design of educational interventions and awareness campaigns that independently inform about nutritional benefits and empower consumers, allowing them to make informed and healthy choices.

## 1 Introduction

Over time, there have been significant changes in food consumption patterns. Health has become one of the most critical factors influencing food choices (1–4). However, individuals have varying attitudes toward food and dietary behaviors, resulting in different food patterns and preferences (5–7).

People’s willingness to consume healthy brand-name foods can vary widely due to some factors, including personal preferences, purchasing power, health awareness, nutritional information and labeling, lifestyle, and dietary choices, price and availability, brand reputation, and cultural and social trends (8). Previous literature recognized a significant association between the willingness to consume foods from healthy brands with nutritional information and labeling since having clear and transparent information about the ingredients and nutrition of the product or food can influence purchasing decisions (9, 10). Brands that emphasize health attributes can attract health-conscious consumers (11, 12). In this sense, the intention to consume healthy branded products is a complex and multifaceted phenomenon involving personal and contextual factors. Food companies often adapt to these trends and adjust their marketing strategies to attract consumers looking for healthier options since studies ensure that social influence affects food intake and choice (13).

On the other hand, it is worth mentioning that nutritional literacy is considered one factor contributing to good practices in maintaining an adequate diet; that is, it provides the community with valuable information about the risks associated with malnutrition (14). Nutrition literacy is essential to promote health and well-being at individual and societal levels. Promotes disease prevention, weight control, healthy eating habits, and consumer empowerment, thus creating a healthier society that recognizes the importance of a balanced diet (15, 16).

Therefore, the willingness to consume foods from healthy brands and nutritional literacy are key aspects that are vital in promoting a balanced and healthy diet (11, 17, 18). Together, they are committed to building a healthier society where consumers can make informed decisions that benefit their well-being and society (8, 16).

Continuing education on healthy eating is critical to addressing nutrition-related public health issues and promoting healthier lifestyles. However, very little has been written about the willingness to consume foods from healthy brands (5, 19–21). One of their studies refers to how emotional regulation influences food choice. When people reduce incidental negative emotions caused by reappraisal, they prefer foods from healthier brands more than when they let their feelings decide (21).

The theory of planned behavior (TPB) has been widely used to examine various aspects of people, such as to predict organic food adoption behavior (22), dietary behaviors (23), also to predict sugar intake (24), the intention to adopt a healthy diet (25) and fast food consumption (26); furthermore, its application has also been transferred to other contexts such as oral health behaviors (27), improving medication adherence (28), teaching complementary medicine (29), pro-environmental behavioral intention (30) and understanding Facebook user behavior (31). In this sense, the TPB is used to understand and predict human behavior, particularly in decision-making associated with planned actions or behaviors (22). TPB has been used in various fields, including health, psychology, marketing, and management. The application of the TPB is crucial because it provides a solid and practical theoretical framework for understanding, predicting, and changing people’s behavior (32). By understanding the motivations and perceptions of decisions, professionals can develop more effective strategies to encourage desired behaviors and increase the effectiveness of interventions in different fields of action (25).

After a diligent review of the background above, there has been a growing interest in continuing to study these topics for academics and professionals in the business and health sectors. Although scientific evidence validates that among the topics of study, the theory of planned behavior has caused the most significant interest. However, bibliometric indicators reveal the ten countries that most disclose their scientific results: the USA, China, United Kingdom, Australia, Iran, Malaysia, Canada, Taiwan, Indonesia, and South Korea. The same ones have applied their study to various areas, sectors, and populations, such as business, social sciences, medicine, psychology, environmental science, and engineering. When discerning the scientific dissemination by country, the studies carried out in the Peruvian population have been found to be very limited. That is, very little scientific literature can provide support and guidance for future studies in this context. Given the prevalence of diseases and poor eating practices related to nutrition, this research aims to fill the knowledge gap and provide a valuable contribution to the academic community and professionals of the sectors involved. In this sense, the purpose of the research is to build a predictive model through an empirical study to examine the influence of Nutritional Literacy (NL) on attitude (ATT), subjective norm (SN), and perceived behavioral control (PBC), as well as determine the influence of three variables of the theory of planned behavior (TPB) on the willingness to consume (WCHBF) for healthy products in the Peruvian market.

## 2 Theoretical background

### 2.1 Research variables

#### 2.1.1 Nutritional literacy

Nutritional literacy was first established in 1995 to assess awareness of nutrition labels among healthy individuals in Canada (14, 33, 34). Nutrition literacy is an individual’s ability to understand, evaluate, and effectively use nutritional information (35, 36). It involves gaining knowledge about the principles of healthy eating, interpreting nutritional information in foods, and making informed decisions about diet and nutrition (37, 38). Nutritional literacy is crucial in promoting the health and well-being of consumers and can help prevent diet-related diseases such as obesity and diabetes. Furthermore, it can foster healthy eating habits and make informed dietary decisions (14, 37).

Critical nutrition literacy involves skillfully evaluating and scrutinizing nutrition information and guidance and being motivated to address nutritional obstacles from individual, societal, and global perspectives (17). In this context, there has been a noticeable increase in the interest and focus on nutritional literacy (33). Nutritional educational programs have been proven essential in formal and informal settings such as schools, universities, and communities. These programs aim to educate consumers about a balanced and healthy diet. The programs also teach them how to interpret nutritional information and make informed decisions about their diet and nutrition (34, 39, 40). Clear and understandable food labeling is essential. It helps consumers understand nutritional information and make better choices about their diet. Promoting healthy foods is also crucial (41, 42). Marketing campaigns, strategic discounts in stores and supermarkets, and offering nutritious options in restaurants and cafes all encourage healthier eating habits (43, 44). Quick access to healthy foods is also important. This strategy ensures that fruits, vegetables, whole grains, and lean proteins are available and accessible in stores and supermarkets (45, 46). It also encourages the creation of community gardens and farmers markets to promote a nutritious eating environment (47). Finally, collaboration with the food industry is essential. This cooperation seeks to develop and promote healthy foods, reduce the sugar, salt, and fat content in processed foods, and promote transparent and responsible food marketing (38, 48).

#### 2.1.2 Willingness to consume healthy brand food

In today’s context, consumer behavior strongly favors foods that contribute to health and well-being, driven by consumers’ enhanced access to tools empowering informed decisions about their nutritional choices (49, 50). Consumers’ willingness to choose and consume healthy branded foods is related to their attitude toward foods perceived as healthy or beneficial to their health (51–53). Numerous elements, including brand perception, nutritional information, cost, and personal preferences, can affect one’s attitude toward preferring this food (54–57). According to previous studies, a brand’s perceived healthiness, product quality, and understandable nutritional information availability all impact consumers’ willingness to buy and consume healthy branded foods (42, 58–60). Additionally, socio-economic factors like income level, education, and occupation can also impact the willingness to consume brand-name healthy foods (61, 62).

In addition, it is advisable to use nudge marketing tools to promote consumers’ willingness to choose and consume healthier brand foods (63–65). Nudge marketing tools refer to marketing strategies influencing consumers’ readiness to select and consume more nutritious food products from specific brands. These tools may include visually appealing and informative nutritional labels, strategic product placement in retail environments, and promotional offers or discounts for healthier options (66, 67). The aim is to encourage consumers to make healthier food choices willingly, positively impacting their overall dietary habits (63, 68). This concept is particularly relevant in increasing awareness and concern about health and nutrition, where marketers seek effective and ethical ways to promote healthier brand foods without infringing upon consumers’ freedom of choice (65, 69).

#### 2.1.3 Theory of planned behavior

The Theory of Planned Behavior (TPB) is a psychological framework that helps predict and understand human behavior (70). It is commonly used to predict purchasing and consumption behaviors (70–73). TPB proposes three main factors influencing a person’s behavior: attitude toward the behavior, subjective norm, and perceived behavioral control (70, 74).

First, consumer attitude towards the behavior refers to how a person evaluates the behavior they are considering (52, 75). According to Ajzen and Madden (76) “attitude” denotes the degree to which a person has a favorable or unfavorable opinion or evaluation of an action or behavior. People who hold a better attitude towards a particular behavior are more likely to have the intention to perform that action (77).

Subjective norms refer to a person’s social pressure to perform or not perform a behavior (70, 75, 78). According to Madden et al. (79), an individual’s close friends are the primary reference group influencing subjective norms. Subjective norms also refer to the positive or negative judgments and perceptions of individuals or groups that believe the individual should perform a particular action (78).

Lastly, perceived behavioral control is a term used to refer to the ease or difficulty with which an individual can carry out a behavior (74, 79). A combination of control beliefs and an individual’s perceived power significantly predict their intention (80). In other words, PBC refers to an individual’s ability to control and perform a specific action. This concept is crucial as it ensures that individuals can easily carry out their intended actions, leading to successful behavior change (74).

### 2.2 Conceptual model and research hypothesis

#### 2.2.1 Influence of nutritional literacy on the theory of planned behavior

Previous studies have indicated that higher nutritional literacy influences consumers to have more positive attitudes towards healthy eating (49). According to Ramdam et al. (81) having more excellent nutrition knowledge is directly linked to more positive attitudes toward selecting healthy foods. Furthermore, Tian et al. (82) point out that labels such as educational materials and food promotions provide objective information to consumers when purchasing a healthy diet. This situation is attributed to the fact that individuals with better nutritional literacy tend to understand the benefits of a balanced diet, which positively influences their attitudes toward healthy eating and, therefore, towards healthy food brands. Similarly, Miller and Cassidy (83) understand nutritional information might be crucial in making dietary decisions through various means. This influence may extend beyond reliance on explicit food label details, encompassing direct impacts on food choices or shaping attitudes and beliefs. Considering these precedents, the hypothesis posits that:

*H1*: Nutritional Literacy (NL) positively influences consumer attitudes toward healthy brand food consumption.

According to previous research, people’s understanding of nutrition can affect their personal beliefs about eating habits (84, 85). Nutritional labels impact the consumer and the social environment, especially when consuming healthy products. For instance, when dining out, the people who matter to the consumer prefer to select a menu item that provides nutritional information (86). Similarly, Sousa et al. (87) found that customers’ intentions to purchase products with nutritional labels were significantly related to their subjective norms, indicating that consumers are influenced by their peers when using food labels to select healthy foods. Considering these antecedents arises the hypothesis that:

*H2*: Nutritional Literacy (NL) positively influences Subjective Norms (SN) for healthy brand food consumption.

Previous studies highlight the significant impact of food literacy on consumers’ perceived control and informed decision-making regarding their dietary choices. Sousa et al. (87) revealed a strong correlation between food literacy and perceived behavioral control, indicating that understanding nutrition information on food labels empowers individuals, amplifying their control over food choices. Similarly, Trieste et al. (88) observed that individuals knowledgeable about nutrition exhibit heightened attention toward nutritional aspects in products, facilitating informed decisions in food consumption. Begley et al. (89) further supported these findings, proposing that promoting food literacy programs can instigate positive shifts in eating behavior and facilitate well-informed decision-making in dietary choices. Taking into account these antecedents, the hypothesis proposes that:

*H3*: Nutritional Literacy (NL) positively influences Perceived Behavioral Control (PBC) for healthy brand food consumption.

#### 2.2.2 Influence of the theory of planned behavior on willingness to consume healthy food

The theory of planned behavior has been fundamental in understanding consumer food decisions (90–92). Based on the idea that attitudes, perceived social norms, and perceived behavioral control influence a person’s intentions and behaviors, this psychological theory has been successfully applied in numerous studies focused on consumers’ food choices (91–97).

Previous studies have established a strong association between consumers’ attitudes and their intention to consume healthy food (93–95, 98–100). For instance, Roseman et al. (101) highlight the importance of the connection between consumers’ attitudes toward food and their intention to buy it. Creating a positive perception among consumers is crucial, as it can significantly affect their buying and consumption behavior (102–104). Küster-Boluda and Vidal-Capilla (98) stated that consumers’ favorable attitudes toward functional foods significantly impact their likelihood of consuming them. Similarly, Khan et al. (105) emphasize that a positive consumer attitude toward an organic product can generate purchase intentions and result in an actual purchase. There is a correlation between consumer attitude and the intention to consume health-oriented products. Grounded in these precedents, the hypothesis posits that:

*H4*: Attitude (ATT) positively influences the consumers’ willingness to consume healthy brand food (WCHBF).

Prior research has established that consumers’ subjective perceptions of the social environment and expectations significantly impact their purchasing and consuming healthy brand foods (94, 96, 106–108). This is mainly because societal trends toward healthier eating and increased individual responsibility for personal well-being have strongly impacted consumers’ choices to buy organic foods (90, 109). For example, Lim and Goh (93) emphasized how social norms and the reference opinions of other individuals positively impact the consumer’s inclination toward purchasing healthy drinks. This finding highlights the relevance of considering social influences in formulating effective strategies to encourage the acquisition of organic products. According to Teng and Wang (108) people who are significant in a consumer’s life can influence their intention to purchase organic products. If consumers perceive that those important to them have a favorable or unfavorable view of organic food, it can affect their purchase intentions towards organic food. Similarly, Agnoli et al. (97) affirmed a high association between wine consumption and social referents. They observed that close friends and partners considerably influenced subjective influence, implying that they are essential in controlling drink consumption. Based on these backgrounds, the hypothesis suggests that:

*H5*: Subjective Norms (SN) positively influence the consumers’ willingness to consume healthy brand food (WCHBF).

Previous research has shown that people are more likely to consume healthy brand foods when they control their behavior (53, 90, 92, 96). A study conducted by Ham et al. (96) found a significant correlation between an individual’s intention to purchase organic food products and their sense of control over their actions. This emphasizes the importance of an individual’s confidence in making informed choices regarding their food preferences. Another study by Giampietri et al. (110) revealed that consumers are more likely to buy their preferred food items when they feel they have greater control over their behavior. However, people’s perceptions of control over their actions may vary in different circumstances. This could be linked to how much power a consumer has over what they choose to purchase and consume. As a result, it can affect a consumer’s willingness to buy organic food (53). Based on these antecedents, the hypothesis suggests that:

*H6*: Perceived Behavioral Control (PBC) positively influences the consumers’ willingness to consume healthy brand food (WCHBF).

**Figure 1:**
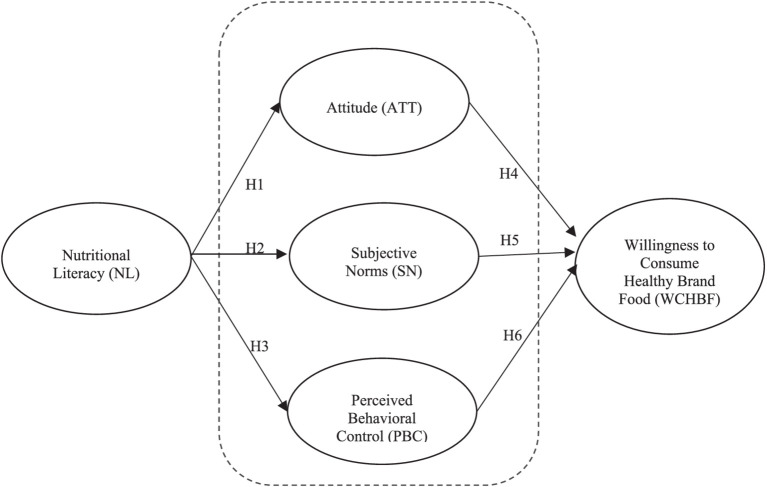
Conceptual model.

Considering the hypotheses mentioned above, the ensuing conceptual model of the study can be visualized, as depicted in Figure 1. Additionally, the advanced hypotheses and their associated constructs have been briefly outlined in tabular form and are available for reference in Appendix A.

## 3 Methods

### 3.1 Context and method

This article aimed to build a predictive model through an empirical study to examine the influence of Nutrition Literacy (NL) on attitude (ATT), subjective norm (SN), and perceived behavioral control (PBC), as well as determine the influence of the three variables of the theory of planned behavior (TPB) on the willingness to consume (WCHF) for healthy products in the Peruvian market. The study used a quantitative, non-experimental, and cross-sectional design approach, using a self-administered questionnaire (111).

### 3.2 Sample and procedure

Different authors recommend different sample sizes. The optimal sample size recommended by some authors should be more than 100 subjects, and the minimum acceptable is at least five times the number of items to be analyzed; however, they suggest that ten times the number of items to be interpreted would be more acceptable (112, 113). Considering these recommendations and given that the instrument of the present study is composed of 27 essential items and three socio-demographic items, a minimum sample of 300 subjects was established. In the end, a sample of 482 participants was obtained, which is above the minimum required sample.

Taking into account that, according to the last Peruvian census, the Lima population is made up of 9,485,405, where women predominate (114) and under the support established by the researchers by stating that young people (5, 115–118) are the ones who tend to consume healthy products compared to adults, considered more traditional, the sample was based on university students. Non-probabilistic convenience sampling was applied to collect data for this research (119). It should be noted that this study was approved by the Ethics Committee of the EPG of the Universidad Peruana Unión (2023-CE-EPG-00043) and was conducted in accordance with the ethical standards of the Declaration of Helsinki. An online survey was carried out through the Google form, the link of which was shared through the official social networks of a private university, an institution where an adequate lifestyle is promoted through healthy eating and which also has the Unión industry within its facilities, applied during the period from July to December 2023 in the city of Lima, Peru. The investigation focused on consumers who stated they were consumers of the Unión brand (whose value proposition is the sale of healthy foods). Participants had to be of legal age, from 18 years onwards. Men and women were invited to participate in the survey; however, the response rate differed (see Table 1). To participate in the survey, each consumer must provide informed consent (Under the premise: ‘*I acknowledge that by completing this questionnaire, I am giving my consent to participate in the study*’). To do so, they were previously informed that their participation was voluntary, that the data collected would be analyzed anonymously, and that they would be used exclusively for academic and research purposes. Nearly 800 Peruvian consumers were invited to this survey, 482 correctly completed questionnaires were answered, and they were considered suitable to be considered in the analysis of this document. Of them, the most significant number of participants were between 18 and 24 years old (84%), were female (65.1%), and their civil status was single (95.2%) (see Table 1).

**Table 1:** Socio-demographic data of the sample (*n* = 482).

Category		Frequency	Percentage
Age range	18–24	405	84.0
	25–34	62	12.9
	35–58	15	3.1
Sex	Male	168	34.9
	Female	314	65.1
Civil status	Married	23	4.8
	Single	459	95.2

### 3.3 Measures

To evaluate the Nutritional Literacy (NL) variable, this study applied the short 11-item scale developed by Vrinten et al. (120). To evaluate the variables of the theory of planned behavior, adaptation was proposed by Kumar et al. (121), where Attitude, Subjective Norm, and Perceived Behavioral Control have three items each, and the willingness to consume foods from healthy brands has seven items (Appendix B). All items are evaluated using a 5-point Likert-type scale, where “1” means “Strongly disagree” and “5” means “Strongly agree.” The digital questionnaire was divided into two sections. The first section presented the 27 items already mentioned, and the second section was composed of questions related to socio-demographic data such as age, sex, and civil status.

### 3.4 Analysis of data

Two statistical software packages were used to analyze the data: IBM SPSS version 22 was used to analyze the respondents’ demographic data, shown in Table 1. Tests for discriminant validity, convergent validity, and reliability were carried out to assess the measurement model (122). Smart-PLS version 4.0 was used to test the conceptual model (see Figure 1) using a two-step approach involving measurement model evaluation and structural model evaluation (122). The PLS-SEM partial least squares method was used to test the hypotheses. PLS-SEM is a comprehensive multivariate statistical analysis approach that includes structural and measurement components to simultaneously examine the relationships between each of the variables in a conceptual model, which has the characteristic of multivariate analysis, i.e., it involves a number of variables equal to or greater than three (119). In addition, PLS-SEM was used in the present study because it facilitates the construction of theories (123).

The significance of the path coefficients (*p*-value and *t*-value) was sought to evaluate the structural model. The coefficient of determination (*R*^2^) was used to measure the predictive relevance of the structural model. Finally, the overall model fit was measured using the root mean square residual (SRMR). It is noteworthy that behavioral scholars have praised the application of PLS-SEM in interdisciplinary research (124).

## 4 Results

Before carrying out the model analyses, the exploratory data analysis was previously carried out with the SPSS-22 software and it was detected that there were no inconsistencies and no outliers, so there was no need to transform the data. Furthermore, according to the contributions of Professor Gaskin, since it is a Likert scale, there are no atypical values, since the participant responds at the extreme (1 or 5), which is why it does not represent a representative atypical component (125).

The application of the PLS-SEM software is carried out through two stages: (1) evaluation of the measurement model and (2) evaluation of the structural model. The first stage evaluates the validity and reliability of the measurement model, and the second evaluates the structural model, which addresses the relationships between the constructs (126, 127).

### 4.1 Evaluation of the structural model

To evaluate the internal consistency of the measurement model, it is necessary to evaluate the convergent validity and reliability of the construct. Convergent validity is acceptable if the loading of each indicator is greater than 0.7 (123). The composite reliability (CR) should be above 0.70, and the average variance extracted (AVE) should be above 0.5 (126, 127). Cronbach’s alpha coefficient should be greater than 0.7. The factor tends to be similar to CR values when factor-based algorithms are used (128). Table 2 shows that all the loadings of the 27 items of this construct had a value greater than 0.7 (except NL10 and NL11; however, together, they meet the reliability). Likewise, all the constructs’ Alpha and CR values were more significant than 0.80, and all the AVE values were more significant than 0.50. Therefore, the convergent validity of the measurement model was excellent. The skewness and kurtosis of the data distribution are also shown, and it is noted that all values are below +/−1.5, which indicates slight variations from the normal and, consequently, results suitable for carrying out factor analysis (129). Although the method used for statistical analysis in this study does not require compliance with normality, these data provide information about the distribution of the data.

**Table 2:** Results of the measurement model.

Construct	Items	Skewness	Kurtosis	loadings	(*α*)	C.R.	AVE
Attitude (ATT)	ATT1	−1.025	0.943	0.961	0.965	0.965	0.934
	ATT2	−1.068	1.068	0.970			
	ATT3	−1.127	1.086	0.969			
Nutritional Literacy (NL)	NL1	−0.608	0.503	0.742	0.926	0.933	0.576
	NL10	−0.571	0.012	0.659			
	NL11	−0.595	0.209	0.682			
	NL2	−0.580	0.290	0.815			
	NL3	−0.576	0.116	0.788			
	NL4	−0.544	0.264	0.817			
	NL5	−0.589	−0.073	0.752			
	NL6	−0.506	0.101	0.800			
	NL7	−0.511	0.224	0.720			
	NL8	−0.297	−0.442	0.786			
	NL9	−0.226	−0.357	0.766			
Perceived	PBC1	−0.635	0.370	0.898	0.852	0.857	0.772
Behavioral Control (PBC)	PBC2	−0.504	−0.135	0.878			
	PBC3	−0.421	−0.216	0.859			
Subjective Norms (SN)	SN1	−0.724	0.549	0.926	0.931	0.931	0.878
	SN2	−0.884	0.937	0.942			
	SN3	−0.850	0.649	0.944			
Willingness to Consume Healthy Brand Food (WCHBF)	WCHB1	−0.764	0.358	0.813	0.931	0.935	0.706
	WCHB2	−0.574	−0.183	0.861			
	WCHB3	−0.748	−0.020	0.801			
	WCHB4	−0.771	0.214	0.868			
	WCHB5	−0.701	0.033	0.874			
	WCHB6	−0.723	−0.038	0.843			
	WCHB7	−0.712	−0.110	0.817			

Cronbach’s alpha (*α*) for all variables is >0.8, the composite reliability (CR) is >0.70, and the mean–variance extracted (AVE) is >0.50, indicating the model’s significant validity.

The Fornell–Larker criterion was used to evaluate discriminant validity, so the square root of the AVE of each construct was calculated, which had to be greater than the highest correlation between the construct and other constructs in the model (126, 127). Table 3 shows that all bold diagonal values are more significant than the correlations. Therefore, the measurement model meets all the necessary assumptions to continue evaluating the structural model.

**Table 3:** Discriminant validity (Fornell–Lacker criterion).

	ATT	NL	PBC	SN	WCHBF
Attitude (ATT)	**0.967**				
Nutritional Literacy (NL)	0.400	**0.759**			
Perceived Behavioral Control (PBC)	0.687	0.522	**0.879**		
Subjective Norm (SN)	0.824	0.447	0.696	**0.937**	
Willingness to Consume Healthy Brand Food (WCHBF)	0.555	0.458	0.562	0.599	**0.840**

The square root of AVEs is shown diagonally in bold.

### 4.2 Evaluation of the structural model

**Figure 2:**
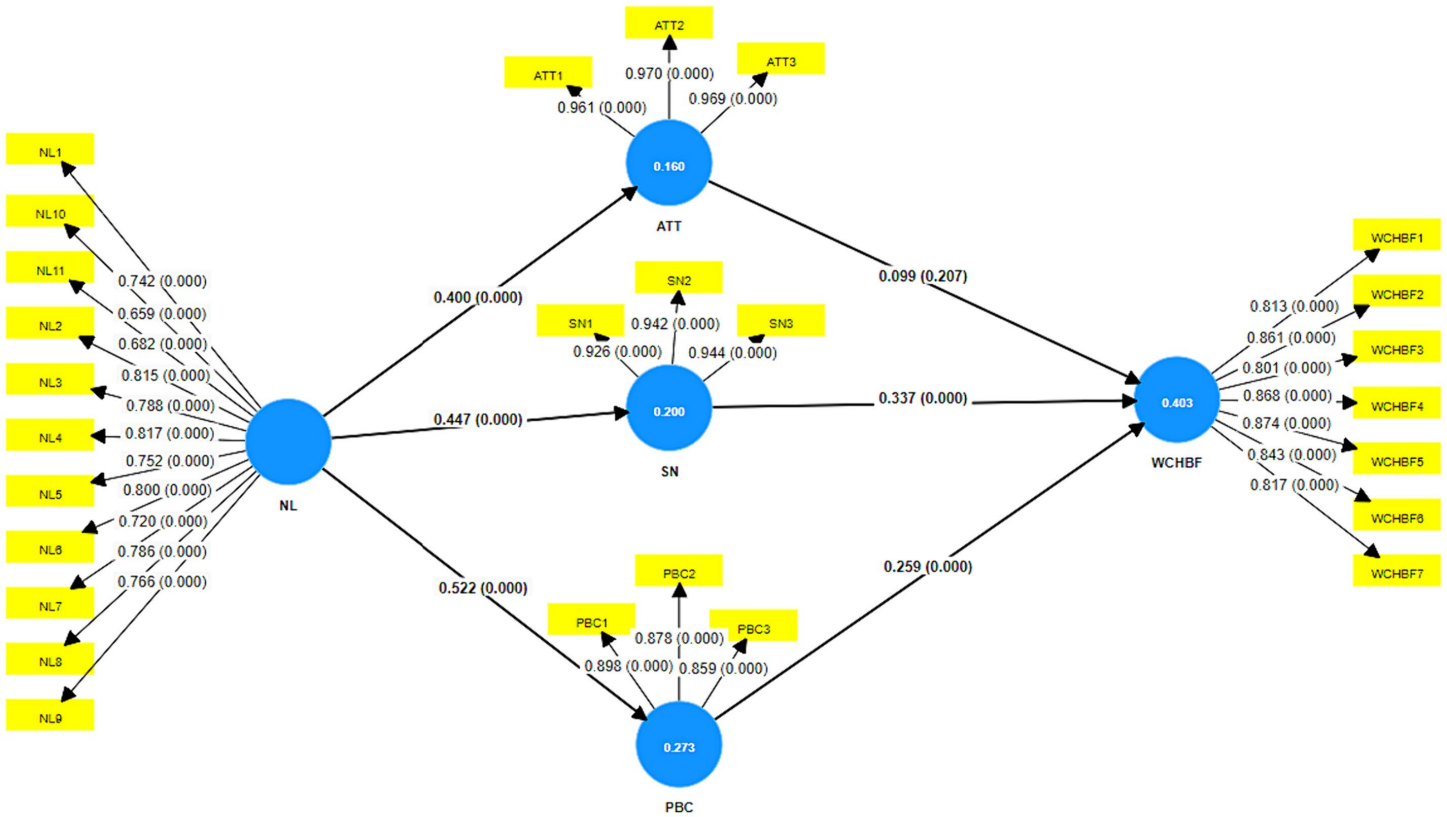
Structural model. PBC, perceived behavioral control; NL, nutritional literacy; ATT, attitude; WCHBF, willingness to consume healthy brand food; SN, subjective norms.

After completing the discriminant, convergent, and reliability tests, the structural model was evaluated using the PLS bootstrapping algorithm with a complete result, a subsample of 5,000, and a one-tailed *t*-test at a significance level of 0.05%. The outcomes of the structural model with the path coefficient, which ought to be a value between −1 and +1 (111), are displayed in Figure 2.

Chin (130) suggests values of 0.67, 0.33, and 0.19 as substantial, moderate, and weak measures of *R*, respectively. In behavioral studies, a value of 0.2 for *R*^2^ is acceptable (127, 131). The present work’s *R*^2^ coefficients for ATT, SN, PBC, and WCHBF were 0.160, 0.200, 0.273, and 0.403, respectively. That is, the *R*^2^ values were acceptable, except for ATT, which is weak. Therefore, the values show that the variables in the present study explain an acceptable percentage of the variance of the WCHB. The overall model fit was measured by the root mean square residual (SRMR), resulting in a value of 0.054 for this indicator, which was below the recommended threshold value of 0.080 (126, 127), thus confirming the model fit.

The hypothesis tests and the evaluation of the path coefficients can be seen in Table 4. The results show that NL has a positive and significant effect on ATT, SN, and CBP, which supports hypotheses H1, H2, and H3. The results show that SN and PBC positively and significantly affect WCHBF, which supports hypotheses H5 and H6. This model indicates that ATT does not impact the WCHBF, so H4 is not accepted.

**Table 4:** Hypothesis testing.

Hypothesis		Original sample (O)	Sample mean (M)	Standard deviation (STDEV)	T statistics (|O/STDEV|)	*p*-values	Decision
H1	NL > ATT	0.400	0.401	0.046	8.661	0.000	Supported
H2	NL > SN	0.447	0.448	0.046	9.805	0.000	Supported
H3	NL > PBC	0.522	0.524	0.041	12.863	0.000	Supported
H4	ATT > WCHBF	0.099	0.100	0.078	1.262	0.207	Rejected
H5	SN > WCHBF	0.337	0.334	0.083	4.089	0.000	Supported
H6	PBC > WCHBF	0.259	0.262	0.060	4.286	0.000	Supported

### 4.3 Invariance analysis and moderating effects: sex

To determine whether there is a difference in the willingness to consume healthy brand foods between male (34.9%, *n* = 168) and female (65.1%, *n* = 314) consumers, it is necessary first to perform the invariance analysis. In Table 5, the MICOM-STEP2 analysis is shown, which allows for the verification that there is no construct difference between men and women; that is, it allows for the verification of the construct invariance between both groups. In this sense, since the *p*-values are not significant (*p* > 0.5), it is concluded that there is invariance in the data collection of both groups, both men and women (132), so it is possible to continue with the analysis of the differences between both groups.

**Table 5:** Invariance analysis.

Construct	Original correlation	Correlation permutation means	5.0%	Permutation *p*-value
ATT	1.000	1.000	1.000	0.892
NL	0.999	0.999	0.998	0.339
PBC	0.999	1.000	0.999	0.215
SN	1.000	1.000	1.000	0.988
WCHBF	1.000	1.000	0.999	0.457

Using the Multigroup Bootstrap Analysis (Bootstrap MGA), we proceeded to verify the difference in the results of the contrast of the hypotheses between men and women, after performing an analysis of 5,000 samples, the results show that none of the *p* values is less than 0.05, therefore (132), There is no significant difference between men and women in the contrasts of the hypotheses raised in this study (see Table 6).

**Table 6:** Bootstrap MGA.

Hypothesis		Difference (Female − Male)	1-tailed (Female vs Male) *p* value	2-tailed (Female vs Male) *p* value
H1	NL > ATT	−0.081	0.816	0.368
H2	NL > SN	−0.093	0.850	0.300
H3	NL > PBC	−0.045	0.715	0.571
H4	ATT > WCHBF	0.121	0.251	0.501
H5	SN > WCHBF	−0.229	0.883	0.234
H6	PBC > WCHBF	0.051	0.349	0.698

## 5 Discussions and conclusions

Nutritional literacy is a topic that has gained significant momentum within the scientific community, and research involving health care has aroused a high interest in contributing to a healthy lifestyle (133, 134). Although applying strategies that allow a change in consumer purchasing behavior toward healthy brands is a challenge, there is research that supports the findings of this study by supporting that there is a fundamental element that allows a positive change in consumer attitudes regarding the consumption of foods from healthy brands, this being nutritional literacy (135). Another study that defends the results of this research is based on the health behavior model, which states that human beings can change their habits that involve health as long as they are influenced by support for medical care. When people feel this support, they experience positive results that lead to maintaining good health behavior (135). However, the lack of nutritional information could generate severe health problems since consumers with this deficiency tend to minimize the value of healthy brands; therefore, it is necessary to use assertive measures so that consumers choose to purchase foods from healthy brands (136, 137). In general terms, antecedents that support this investigation have been found. Regardless of acquiring knowledge regarding a daily diet, nutritional literacy triggers an essential influence for a consumer to adopt healthy food consumption habits.

Furthermore, another result of this research proves that nutritional literacy exerts an essential influence on subjective norms for the consumption of healthy foods. Makiabadi (138) establishes that a person’s knowledge shapes the behavior of human life. That is to say, the greater the understanding of information, the more subjective norms point to a better decision when purchasing their food. Another antecedent that gives essential support to these findings is the theory of planned behavior. According to the research, this theory describes eating behavior, where subjective norms are influenced by nutritional literacy (139). Furthermore, it has been identified that the intention to change behaviors linked to nutrition is significantly influenced by subjective norms, with personal perceptions being a response to the consumer’s prior knowledge, translating this fact into further support that affirms that nutritional literacy influences subjective norms (140). In this context, the results of this study are reinforced, specifying that nutritional literacy influences subjective norms, constituting this fact as an opportunity to provide further strength to interventions aimed at promoting the consumption of healthy brand foods.

The third hypothesis that proposed investigating whether nutritional literacy positively influences the perceived behavioral control for consuming healthy brand foods has been rejected. To further support this finding, research has found that in the event of an inadequate state of health, nutritional literacy allows an individual to understand and raise awareness to follow nutritional advice, representing a change in perceived behavior (34, 141). A similar study addressed this behavior in adolescents, stating that this population has greater ease and access to nutritional information when required; thus, when an individual maintains a higher level of nutritional literacy, he or she maintains better control of perceived behavior (141). This means that every individual with a level of nutrition knowledge has a more fantastic option of making informed decisions and ratifying his or her decision, even when external pressures seek to be against healthy food choices. Furthermore, other research that supports this study indicates that nutritional literacy is an indicator that influences behavior and decisions regarding food consumption (142, 143).

Within the findings, it has been identified that attitude does not influence the willingness of consumers to consume healthy branded foods. It is that even though traditionally, it has been considered that attitude plays a determining role in the formation of individual behaviors and consumption decisions (144, 145). Within the study population, the choice of healthy foods is not a determinant in predicting the willingness to consume healthy branded foods, so it is claimed that other factors may have a more marked influence. While Renwick and Smith (146) refer that the attitudes of individuals can address a series of decisions, the same that with repetition becomes a habit, in the case of the Peruvian population, beyond the choice of a healthy brand, other factors intervene in the final decision. Under this context, Perry (147) states that beyond attitudes, knowledge, and skills can influence the final decision.

This study sheds light on the influence of subjective norms on consumers’ willingness to consume healthy branded foods. To better explain this finding, it is necessary to clarify that subjective norms involve “perceived social pressure to perform a particular behavior” (148). In this context, Oktavianus and Bautista (149) support the idea that subjective norms have a high potential to improve an individual’s behavioral intention. For its part, Chen and Fu (150) and Bautista et al. (151) declare that when people harbor bad practices due to some erroneous information, subjective norms are part of an ideal component to correct them. When an individual considers that his environment expects him to perform some specific behavior, the possibility of him opting increases. For addressing the expectations of others. Another result that coincides with the evidence established that the perception of a third person increases the intention for an individual to correct their actions, even more so if they are actions that can correct habits that damage health (152, 153). This means that subjective norms are a point of support since the participation of third parties and the social environment build positive attitudes regarding the consumption of healthy brand foods, which represents forming solid habits regarding a diet from healthy brands. Finally, the results suggest that the influence of nutritional literacy on attitude, subjective norm, and perceived behavioral control is similar between men and women. This indicates that both groups may similarly perceive the importance of nutritional literacy in their purchasing decisions for healthy products.

### 5.1 Implications

This study has addressed consumer behavior from the planned behavior perspective within the theoretical implications. Thus, the proposed theoretical model is part of a robust conceptual structure that allows a clear understanding of the factors involved in decision shopping. Based on the theory, specific strategies that aim to correct consumer behavior regarding the consumption of healthy foods can be addressed. Statistics make an essential contribution to the literature on these topics. Therefore, more research is needed to evaluate the data obtained in this study more broadly.

Now, the results lead to discovering specific practical implications that have to do with technology. In a digitalized world, allowing consumers to be well-informed and achieve nutritional literacy is not an impossible task. Still, it does require that the media disseminate information about it. On the other hand, it is necessary to increase nutritional literacy to ensure a healthier diet in the population; therefore, new government policies on health must be developed to achieve these standards. When analyzing projects and programs related to nutrition in underdeveloped countries, some of these successful programs include multiple behavioral development initiatives that could last over time but require a progressive lifestyle change. In this sense, the results of this study can be translated into recommendations to improve nutritional literacy, nutrition, and the development of good eating habits to avoid health risks and complications (poor quality of life, malnutrition, dietary intake, diabetes, among others).

On the other hand, the Ministries of Health and Education should show a more significant commitment to cooperating for this national purpose. Some practical courses, such as healthy eating and lifestyle programs, are necessary to educate a new generation with a broader vision. Currently, some private educational institutions, in their attempt to join this movement, could be spreading less-than-appropriate eating practices. For this reason, these issues should be addressed with permanent guidance from experts in the field.

This study deepens knowledge about nutritional literacy and the consumption of foods from healthy brands, which would allow the senior management of any organization, the academic community, those responsible for the Ministry of Health, and other public and private organizations to consider reforming food policies and designing strategies to improve the health of more citizens.

Finally, given that there are no significant differences between men and women, marketing strategies related to promoting healthy products could be designed more unisex. Campaigns that highlight the importance of nutritional literacy could effectively target both sexes.

### 5.2 Limitations and future research

The size of the sample and the type of sampling (non-probability by convenience) used in the study do not allow the results to be projected onto the composition of the base population. The study sample was observed to have a significant disproportion in terms of civil status, sex, and age range. Due to this, the study’s findings may not be applicable to other populations or contexts since the sample was biased toward specific socio-demographic characteristics. It is suggested that future studies should attempt to obtain more homogeneous samples to avoid this issue.

On the other hand, the study did not consider some essential socio-economic determinants to describe the profile of the participants, such as educational level, health conditions, occupation, and economic income. This fact is part of one of the limitations of this study, so this research could not be generalized. In this way, it is proposed that future research address the differences in perception of the study variables in a different cultural context to measure the gaps. The study was also limited by the time it took to complete the survey, making some prone to abandoning the questionnaire. Furthermore, another limitation of this research is that it has not been considered whether the sample has received or possesses any level of nutritional literacy since the difference between them may be a research bias, so future research should address the level of nutritional literacy to carry out an analysis that measures the strength of influence of one variable on another.

Finally, although there are no significant differences between men and women in the results of this study, it could be beneficial to explore external factors such as culture, advertising, or social events that may influence men and women differently in relation to eating and health. These factors must be considered for future research, in this way to understand the context fully.

## Data availability statement

The raw data supporting the conclusions of this article will be made available by the authors, without undue reservation.

## Ethics statement

The studies involving humans were approved by Universidad Peruana Unión Ethics Committee. The studies were conducted in accordance with the local legislation and institutional requirements. The participants provided their written informed consent to participate in this study.

## Author contributions

RC-T: Conceptualization, Data curation, Funding acquisition, Investigation, Resources, Validation, Visualization, Writing – original draft. EG-S: Conceptualization, Data curation, Formal analysis, Funding acquisition, Investigation, Methodology, Project administration, Resources, Supervision, Validation, Visualization, Writing – original draft, Writing – review & editing. ME-F: Conceptualization, Funding acquisition, Resources, Visualization, Writing – original draft, Writing – review & editing. DM-L: Conceptualization, Funding acquisition, Investigation, Project administration, Resources, Supervision, Validation, Visualization, Writing – original draft, Writing – review & editing. MV-G: Conceptualization, Funding acquisition, Investigation, Resources, Visualization, Writing – original draft, Writing – review & editing.
