# Supplementary material for: Predicting willingness to consume healthy brand foods using the theory of planned behavior: the role of nutritional literacy
Source: Front Nutr. 2024 Mar 22;11:1353569. doi: 10.3389/fnut.2024.1353569 (PMC11025538; doi:10.3389/fnut.2024.1353569)
Supplement: Supplementary file 1 [file Table_1.docx]

**Appendix A.** Hypothesis Related to the Theoretical Model Proposed

| **Construct /Item from the Model** | **Hypothesis** | **Supported by:** |
| --- | --- | --- |
| Nutritional Literacy (NL) | H1. Nutritional Literacy (NL) positively influences consumer attitudes toward healthy brand food consumption. | Vettori et al. (39)  Ramdam et al. (83)  Tian et al. (84)  Miller and Cassidy (85) |
|  | H2. Nutritional Literacy (NL) positively influences Subjective Norms (SN) for healthy brand food consumption. | Vettori et al. (39)  Sobaih et al. (88)  Sousa et al. (89) |
|  | H3: Nutritional Literacy (NL) positively influences Perceived Behavioural Control (PBC) for healthy brand food consumption. | Sousa et al. (89)  Trieste et al. (90)  Begley et al. (91) |
| Theory of Planned Behavior (TPB) | H4: Attitude (ATT) positively influences the consumers' willingness to consume healthy brand food (WCHBF). | Lim and Goh (95)  Sparks et al. (97)  Küster-Boluda and Vidal-Capilla (100)  Quevedo-Silva et al. (102)  Roseman et al. (103)  Khan et al. (107) |
|  | H5. Subjective Norms (SN) positively influence the consumers' willingness to consume healthy brand food (WCHBF). | Lim and Goh (95)  Ham et al. (98)  Agnoli et al. (99)  Chen (108)  Teng and Wang (110) |
|  | H6: Perceived Behavioural Control (PBC) positively influences the consumers' willingness to consume healthy brand food (WCHBF). | Aliaga-Ortega et al. (92)  Ahmed et al. (94)  Ham et al. (98)  Eyinade et al. (112)  Giampietri et al. (113) |

**Appendix B.** Constructs and items

| **Construct** | **Items** | **Loadings** |
| --- | --- | --- |
| Attitude (ATT) |  | *Buying healthy food...* |
|  | ATT1 | It's a good idea. |
|  | ATT2 | It's important. |
|  | ATT3 | It's beneficial. |
| Nutritional Literacy (NL) | NL1 | I can evaluate whether nutrition information in the media is reliable. |
|  | NL2 | If I have questions about healthy nutrition, I know where to find information about it. |
|  | NL3 | When I search for nutrition information online, I can distinguish between reliable and less reliable websites. |
|  | NL4 | If I have questions about sustainable nutrition, I know where to find information. Examples of sustainable nutrition are organic vegetables, free-range chicken eggs, and fair-trade coffee. |
|  | NL5 | I have the skills to apply nutrition information when cooking. |
|  | NL6 | Advertisements often make a connection between nutrition and health. It is easy for me to judge whether these links are true or not. |
|  | NL7 | I know the basic rules of the Food Triangle. |
|  | NL8 | I can evaluate whether nutrition information is written with the intention of making money, for example, by people who want to sell a product. |
|  | NL9 | I follow nutrition advice from experts. |
|  | NL10 | I discuss nutrition information with an expert. |
|  | NL11 | My diet is based on the latest scientific knowledge. |
| Perceived Behavioral Control (PBC) |  | *To buy healthy food...* |
|  | PBC1 | I have the ability. |
|  | PBC2 | I have the resources. |
|  | PBC3 | I have the time and willingness. |
| Subjective Norms (SN) |  | *They think it's important to buy healthy foods...* |
|  | SN1 | The people I listen to. |
|  | SN2 | The people who are important to me. |
|  | SN3 | My friends and family. |
| Willingness to Consume Healthy Brand Food (WCHBF) |  | *I am willing to buy the following Union brand products...* |
|  | WCHBF1 | Bread (multiseed, special whole wheat flour fortified with iron and B vitamins, bromate-free). |
|  | WCHBF2 | Cookies (special whole wheat flour fortified with iron and B complex vitamins, free of artificial coloring). |
|  | WCHBF3 | Beverages (0% alcohol wine, sugar-free fruit juices, free of artificial colors and flavorings). |
|  | WCHBF4 | Granolas (mix of nuts, seeds, and cereals). |
|  | WCHBF5 | Snacks (Sticks with sesame, chia, kion, garlic, flaxseed, and Omega 9, 6, and 3). |
|  | WCHBF6 | Spreads (grape jam, peanut butter, contains Omega 6 and 9). |
|  | WCHBF7 | Panettone (Fortified with iron, contains a source of fiber and protein, and contains Omega 6 and 9). |
